# A Molecular Dynamics Study on the Interfacial Properties Between Polymer Fibers and Geopolymer Resins

**DOI:** 10.3390/ma18184357

**Published:** 2025-09-18

**Authors:** Yanfeng Wei, Bin Ma, Ligen Lan, Yanqi Chen, Xiaolin Huang, Yankun Huang, Chaosong Chen

**Affiliations:** School of Architecture and Transportation Engineering, Guilin University of Electronic Technology, Guilin 541004, China; 15612203593@163.com (Y.W.); llg554822@163.com (L.L.); cyq1021894456@163.com (Y.C.); hxl-68@163.com (X.H.); 17754548159@163.com (Y.H.); 18948979489@163.com (C.C.)

**Keywords:** polymer fibers, geopolymer resins, interfacial properties, anchoring effect, molecular dynamics

## Abstract

In this study, interface models of different polymer fibers and geopolymer products were constructed and the microscopic action mechanisms behind different polar oxygen-containing functional groups were revealed by analyzing the static structure of the interface and fiber pull-out process. The results show that, compared with polypropylene (PP) fibers, there is a strong hydrogen bond interaction in polyvinyl alcohol (PVA) and polyacrylic acid (PAA) fibers, respectively. After reaching equilibrium, the interlayer spacing of PVA and PAA fibers becomes smaller. At the interface, there are both ionic bonds and hydrogen-bonding connections, and the stability of ionic bonds is greater than that of hydrogen bonds. As the loading rate increases, the shear strength shows an increasing trend. Since the carboxyl of PAA fibers has stronger polarity than the hydroxyl of PVA fibers, the end deformation of the matrix is greater. During the pull-out process, the influence of the loading rate is greater than the anchoring effect of hydroxyl in PVA fibers but lower than that of carboxyl in PAA fibers.

## 1. Introduction

As emerging environmentally friendly inorganic cementitious materials, geopolymers have attracted significant attention because of their low energy consumption and low CO_2_ emissions [1]. The main reaction product of geopolymers is sodium aluminosilicate hydrate (NASH). Compared with cement hydration products, NASH contains more aluminum oxygen tetrahedra (AlO_4_) and silicon oxygen tetrahedra (SiO_4_), which are connected by bridging oxygen to form a stable structure. Because of this structural characteristic, NASH offers significant advantages in terms of mechanical properties and durability and has broad application prospects in the building materials field [2]. However, because geopolymers are easily susceptible to shrinkage cracking and brittleness problems, their popularization and application in the engineering field have been limited [3,4].

In order to solve this problem, researchers have actively explored various reinforcement methods, with the introduction of polymer fibers representing a promising strategy [5]. The performance of geopolymers modified by fibers largely depends on the interfacial properties of the fibers and matrix [6,7]. At present, many experimental studies are being conducted at the fiber–geopolymer interface using microstructural identification techniques. Zhang et al. [8] observed the distribution of fibers and interfacial characteristics between the fibers and matrix using optical microscopy and scanning electron microscopy (SEM) techniques. Walbrück et al. [9] combined microscopic testing techniques, such as X-ray diffraction (XRD) and X-ray micro-computed tomography (μCT), to study the microscopic reinforcement mechanisms of geopolymers modified by miscanthus fibers. Shirai et al. [10] used XRD, SEM, and Fourier transform infrared spectroscopy analysis (FTIR) techniques to study the microscopic influence mechanism of polyvinyl alcohol (PVA) fibers on the mechanical properties and interfacial characteristics of geopolymer–ordinary concrete composites. Zhang et al. [11] investigated the interfacial characteristics between polyvinyl alcohol (PVA) fibers and geopolymers by observing the pull-out process under a microscope. It can be seen that traditional experimental methods are associated with many challenges, such as the complexity of specimen preparation, the considerable difficulty of characterizing the interfacial microstructure, and the strict limitations required for the experimental conditions. These factors not only increase the research cost but may also lead to uncertain results because of the instability of the experimental conditions. As a result, it is difficult to fully reveal the microscopic interaction mechanism between the fiber and the matrix.

As an effective computational method, molecular dynamics simulations have been widely adopted to study the interfacial properties of engineering composite materials [12,13,14]. Min et al. [15] established the interface model between carbon fibers and calcium silicate hydrate (CSH), simulated the fiber pull-out process, and investigated the enhancement mechanism of graphene oxide at the carbon fiber–CSH interface. Wang et al. [16] studied the influence of the functional group types and the water content on the interface between graphene oxide and CSH and found that the interfacial bonding strength was enhanced by the interfacial bonds and the roughness of the graphene oxide surface and weakened by water molecules. Wang et al. [7] investigated the effect of the shear strength and microstructure on the interface between fibers and CSH and analyzed the influence mechanisms of polypropylene (PP), PVA, and polyacrylic acid (PAA) fibers. Guan et al. [5] studied the mechanism behind the interfacial interaction between polyethylene (PE) fibers and NASH and suggested that the adhesion was affected by interfacial interactions and mechanical interlocking. At present, most existing studies address the enhancement of cement hydration products by fibers. However, the research on fiber-reinforced geopolymer is still in its initial stage, and there is a lack of research on the influence of polar functional groups.

In this study, the molecular dynamics method is used to investigate the effect mechanism of the fiber polar functional groups on the interfacial properties. To conduct a comprehensive comparative analysis, three fiber models are established: (i) PP fibers without polar oxygen-containing functional groups; (ii) PVA fibers containing hydroxyl groups (-OH); (iii) PAA fibers containing carboxyl groups (-COOH). Then, the different interface models are constructed. By analyzing the static structure of interface and the fiber pull-out process, the microscopic action mechanisms of different polar oxygen-containing functional groups at the interfaces are revealed. This study will provide theoretical guidance for understanding fiber/geopolymer interactions.

## 2. Simulation Methods and Processes

### 2.1. Model Construction

**Figure 1:**
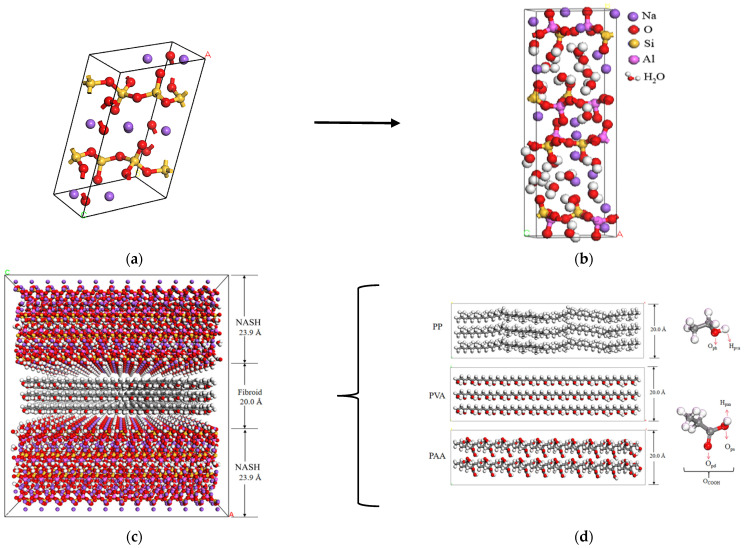
(**a**) Na_2_Si_2_O_5_ glass model; (**b**) unit cell model of the NASH schematic; (**c**) diagram of fiber–NASH composite model structure; (**d**) polymer fiber model (O_ph_ and H_pva_, respectively, represent the oxygen and hydrogen atom of the C-OH group in PVA; O_pd_ and O_ps_, respectively, represent the double-bonded oxygen and single-bonded oxygen of C-COOH in PAA; and H_paa_ represents the hydrogen atom connected to the single-bonded oxygen atom).

Studies have shown that the molecular structure of NASH is similar to that of Na_2_Si_2_O_5_ glass [17,18] (Figure 1a). Therefore, in this paper, the method proposed by Tian et al. [19] is adopted to establish the NASH unit cell model based on the Na_2_Si_2_O_5_ glass model. The steps are as follows: (1) The monoclinic structure of Na_2_Si_2_O_5_ model without water molecules is transformed into a cubic structure, while the relative atomic positions and the model size are kept unchanged during the process. After the transformation, the dimensions of the unit cell in the X, Y, and Z directions are 4.84 Å, 8.13 Å, and 23.90 Å, respectively, and the angles are all 90°. (2) Si atoms of the transformed unit cell are randomly replaced with Al atoms to obtain a NASH model with a Si/Al ratio of 1 [20,21]. (3) Since this atom replacement will result in charge loss, Na^+^ ions are redistributed to ensure that the system is balanced. (4) The structure is optimized, and the model-quenching treatment is carried out (Figure 1b).

To establish the fiber–NASH composite models, firstly, the unit cell of NASH is expanded by a factor of 12, 8, and 2 along the X, Y, and Z directions, respectively. Secondly, the expanded model is cut into two independent upper and lower matrices along the Z direction, and two matrices are moved outward to form a channel with a width of 2.0 nm. Finally, different polymer fibers are inserted into the NASH channel, and then fiber/NASH composite models are obtained with dimensions along the X, Y, and Z directions of 65.06 Å, 58.17 Å, and 67.80 Å respectively, as shown in Figure 1c. Fibers have the same initial velocity and mass to ensure that they have the same initial kinetic energy.

### 2.2. Force Field

The selection of force fields is crucial in molecular dynamics simulations and directly affects the accuracy of simulation results. In addition, a combined empirical force field (CLAYFF + CVFF) has been widely used to investigate the interfacial characteristics between geopolymers and other materials, and with proven effectiveness [22,23]. To study the interfacial properties of fibers and NASH, a combined empirical force field is used in this simulation. For the combination rules for the CLAYFF and CVFF force fields, we referred to the Lorentz–Berthelot method [24], and the parameters were obtained from previous studies [24,25].

The CLAYFF force field is adopted for NASH. The structural properties, dynamic characteristics, and interfacial properties of geopolymer hydration products can be accurately and efficiently simulated using CLAYFF. Furthermore, it has been successfully applied to study the adsorption and diffusion mechanisms of erosive ions in geopolymers [26,27], as well as the desorption behavior at the interface between polymers and clay minerals [28].

The CVFF force field is used to simulate polymer fibers. The potential function of this force field consists of bonding and non-bonding interactions [25] and has been widely used for modeling organic substances [29]. In short, the interactions in fiber–NASH models can be successfully simulated using the combined empirical force field method.

### 2.3. Equilibrium Simulation

LAMMPS software is adopted to carry out molecular dynamics simulations for different fiber–NASH composite models [30]. The equilibrium simulation is carried out according to the method applied by Wang et al. [7]. Firstly, the energy of the model is minimized at the NPT (isothermal–isobaric) ensemble using the method of steepest descent. Then, in order to ensure that the state of the system achieves equilibrium, 1000 ps relaxation is performed at the NPT ensemble, with a time step of 1 fs. The simulated temperature is 300 K, and the pressure is 0 Kpa, controlled by the Nose–Hoover thermostat. The atomic trajectory data are output. In addition, the balance of the system is determined based on the distance change between the centroids of the upper and lower substrates, and the distance of the centroids is constant, indicating that the model has reached equilibrium [31].

**Figure 2:**
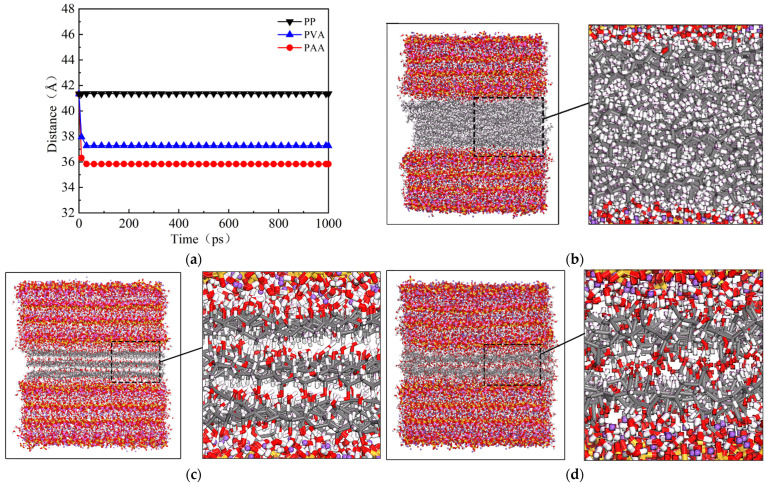
(**a**) Curves for distance between the upper and lower centroids with relaxation time; snapshot images of models after reaching relaxation equilibrium (1000 ps). (**b**) PP-NASH; (**c**) PVA-NASH; (**d**) PAA-NASH.

Figure 2a shows the curves for the distance between the upper and lower centroids of the NASH matrix with the relaxation time. It can be seen that the initial values for the centroid distance are the same for the three models. As the relaxation time increases, the centroid distance of the matrix for PP-NASH is almost unchanged, while the centroid distances of matrices for PVA-NASH and PAA-NASH decrease rapidly and then tend to balance. The centroid distance of the matrix after reaching equilibrium is the longest in PP-NASH, followed by that in PVA-NASH, and PAA-NASH has the shortest.

Figure 2b–d show snapshot images of the models after reaching relaxation equilibrium. The fiber interface width variation in the three models is consistent with the centroid distance of the matrix (Figure 2a). Furthermore, PP fibers are entangled in the layer and between the layers, leading to the originally clear layered structure becoming blurred (Figure 2b). This phenomenon has also been observed in relevant simulation studies [32,33]. Compared with PP fibers, PVA and PAA fibers have polar functional groups, and strong hydrogen bonds are generated between fibers, so their fiber layer spacing becomes smaller after equilibrium [34]. However, since the polarity of the hydroxyl group (-OH) in PVA fibers is weaker than that of the carboxyl group (-COOH) in PAA fibers [35], the layer spacing of PAA fibers is smaller than that of PVA fibers, as illustrated in Figure 2c,d. Smaller spacing usually means that the interface is more tightly bonded, making the hydrogen bonding between molecules more effective and thereby improving the interfacial bonding strength.

### 2.4. Pull-Out Simulation

**Figure 3:**
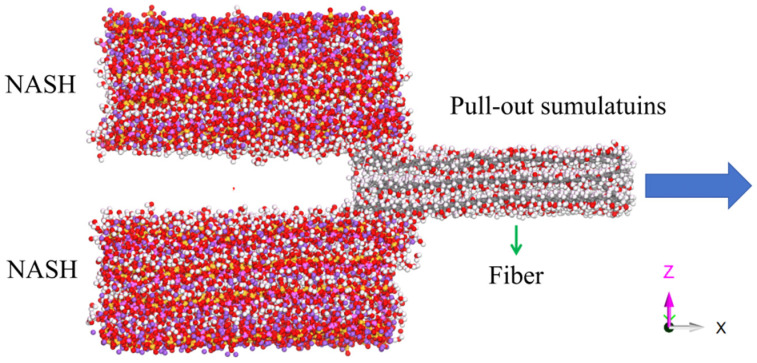
Pull-out simulation of fiber.

To investigate the interfacial interaction, pull-out simulations of different fiber–NASH composite models are conducted along the X direction (the schematic diagram of the principle is shown in Figure 3), and the pulling force and the interface shear strength are also calculated. Firstly, the non-periodic and shrink-wrapping conditions are set in the X direction to eliminate the boundary limitation during the simulation process. Then, the top- and bottom-region (2 Å) atoms of the NASH matrix are fixed. At the NVT ensemble, fibers were pulled out at rates of 0.001 Å/fs, 0.01 Å/fs, and 0.05 Å/fs, with a temperature of 300 K and a pressure of 0 KPa. Finally, the pulling force and displacement curves are elucidated; the pulling force is obtained by calculating the force of all the fiber atoms along the pulling direction.

In addition, the interfacial shear strength is calculated using Formula 1. The specific formula is as follows:

(1) $$\tau =\frac{F_{\max}}{2\mathrm{ab}}$$

where F_max_ is the maximum force of the fiber; a is the length of the box along the X direction; and b is the length of the box along the Y direction. F_max_ can be obtained by calculating the total force applied to all atoms in the fiber part along the X direction.

## 3. Results and Discussion

### 3.1. Interface Static Structure

#### 3.1.1. Atomic Density Distribution

**Figure 4:**
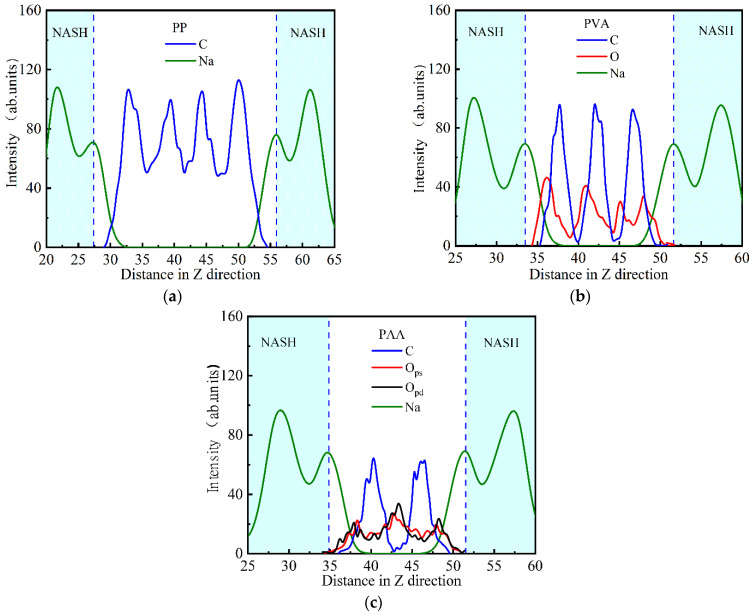
Atomic density distribution of (**a**) PP-NASH; (**b**) PVA-NASH; (**c**) PAA-NASH.

Figure 4a shows the atomic density distribution at the interface of PP and NASH. Na^+^ ions near the surface of the NASH matrix accumulate at 27.33 Å and 55.91 Å from the surface of the PP fiber, and peaks of atomic density are produced. This indicates that the PP fiber has an attractive effect on the Na^+^ ions from the surface of the NASH matrix. Furthermore, the C atom density distribution curve of the PP fiber shows a periodic change with four distinct peaks. Compared with the three-layer PP fiber model of the initial configuration (as shown in Figure 1), the C atom aggregation layer of the PP fiber changes after equilibrium. The periodic trough values on the C atom density distribution curve are significantly higher than zero, indicating that the interlayer structure of the PP fiber becomes blurred after equilibrium. This result is consistent with the entanglement phenomenon in the PP fiber, which can be observed within and between layers in Figure 2a.

Figure 4b shows the atomic density distribution at the interface of PVA and NASH. The C atom density distribution curve of the PVA fiber also shows periodic variation and three obvious peaks, which is consistent with the three-layer PVA fiber model of the initial configuration. Compared with PP-NASH, the periodic trough values on the C atom density distribution curve for the PVA fiber are close to zero, indicating that the PVA fiber has a clear interlayer structure. The peak positions of polar oxygen atoms are closer to the matrix surface than those of C atoms, indicating that the polar oxygen atoms of the PVA fiber have a higher probability of being bridged with the Na^+^ and hydroxyl groups of NASH.

Figure 4c illustrates the atomic density distribution at the interface of PAA and NASH. The C atom density distribution curve of the PAA fiber shows two obvious periodic peaks, which is consistent with the initial configuration. For the PAA fiber, the trough positions on the oxygen atom density distribution curve correspond to the peak positions of carbon atoms, and the peak positions of oxygen atoms correspond to the trough positions of carbon atoms. Moreover, the peak positions of oxygen atoms are closer to the NASH matrix than those of carbon atoms. Compared with PVA-NASH, due to the stronger polarity of carboxyl groups for PAA fibers, there is a stronger bridging effect between PAA fibers and the Na^+^ or hydroxyl groups of NASH. In addition, there are single-bonded oxygen atoms (O_ps_) and double-bonded oxygen atoms (O_pd_) in the PAA fiber. The peak positions on the O_pd_ atom density distribution curve are 37.95 Å and 48.19 Å, respectively, while those for O_ps_ atoms are 38.31 Å and 47.90 Å. It can be seen that O_pd_ atoms are closer to the NASH surface than O_ps_ atoms, indicating that the interfacial bridging effect of O_pd_ atoms is stronger than that of O_ps_ atoms.

#### 3.1.2. Radial Distribution Function

The radial distribution function (RDF) is calculated to study the connection between the fibers and the matrix. It is capable of characterizing the spatial correlation among atoms and investigating the orderliness of substances, which offers an in-depth comprehension of the interaction mechanism. The calculation formula is defined as follows [31]:

(2) $$g(r)=\frac{dN}{4\pi r^{2}\rho }$$

where ρ is the system density; *dN* is the number of molecules with the distance *dr* from the central atom.

**Figure 5:**
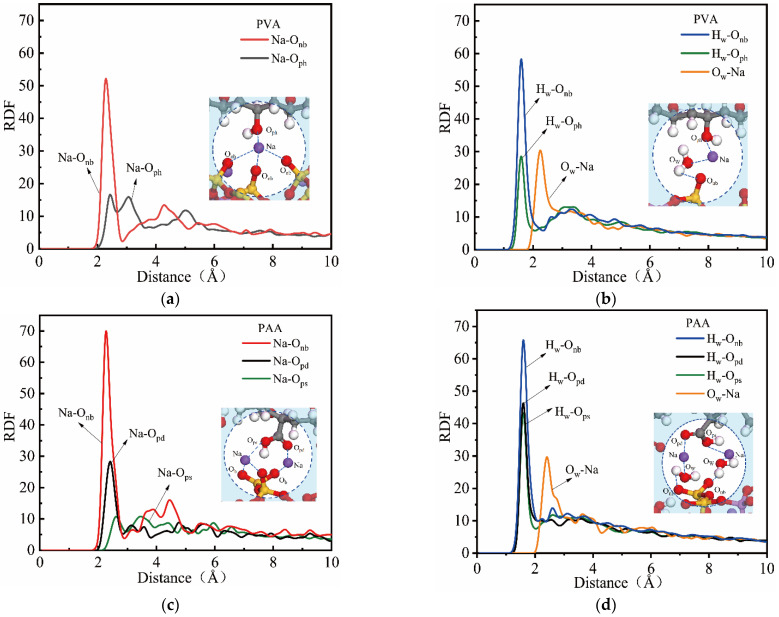
Radial distribution function of (**a**) Na-O bond in PVA-NASH; (**b**) Na-O_w_ bond and H bond in PVA-NASH; (**c**) Na-O bond in PAA-NASH; and (**d**) Na-O_w_ bond and H bond in PAA-NASH (O_nb_ represents the non-bridging oxygen atom in NASH).

Figure 5 shows the RDF of ion pairs in different fiber–NASH models. As shown in Figure 5a, there are obvious peaks at 2.41 Å for the RDF of Na-O_nb_ and Na-O_ph_, indicating the formation of ion pairs between Na^+^ and O_ph_ or O_nb_. O_ph_ in PVA fibers bridges the Na^+^ of the NASH channels, and PVA fibers are connected to the NASH matrix in the form O_nb_-Na-O_ph_.

Figure 5b shows the RDF of Na-O_w_ bonds and H bonds in PVA-NASH. There are obvious peaks in the RDF for Na-O_w_, H_w_-O_nb_, and H_w_-O_ph_, which proves that water molecules also play a bridging role between PVA fibers and the NASH matrix. PVA fibers are connected to the NASH matrix in the forms O_ph_-H_2_O-Na-O_nb_ and O_ph_-Na-H_2_O-O_nb_. In summary, there are both ionic and hydrogen bonds at the interface of PVA and NASH, and the interfacial bonding strength is based on this type of connection.

Figure 5c,d show the RDF of Na-O bonds, Na-O_w_ bonds, and H bonds in PAA-NASH. Compared with the PVA-NASH model, there are six types of interface connection form for PAA-NASH, which are O_nb_-Na-O_pd_, O_nb_-Na-O_ps_, O_pd_-H_2_O-Na-O_nb_, O_pd_-Na-H_2_O-O_nb_, O_ps_-H_2_O-Na-O_nb_, and O_ps_-Na-H_2_O-O_nb_. The RDF peak for Na-O_pd_ is higher than that for Na-O_ps_, and its RDF peak position is also smaller, indicating that the bonding ability of O_pd_ with Na^+^ is stronger than that of O_ps_. This conclusion is consistent with the atomic density distribution (as shown in Figure 4).

#### 3.1.3. Mean Squared Displacement

The mean squared displacement (MSD) is often used to evaluate the atomic migration behavior of a system, to describe the stability of chemical bonds. The specific calculation formula is as follows [31]:

(3) $$\mathrm{MSD}(t)=<{\left|\mathrm{ri}(t)-\mathrm{ri}(0)\right|}^{2}>$$

where ri(0) is the initial position; ri(t) is the position at time t.

**Figure 6:**
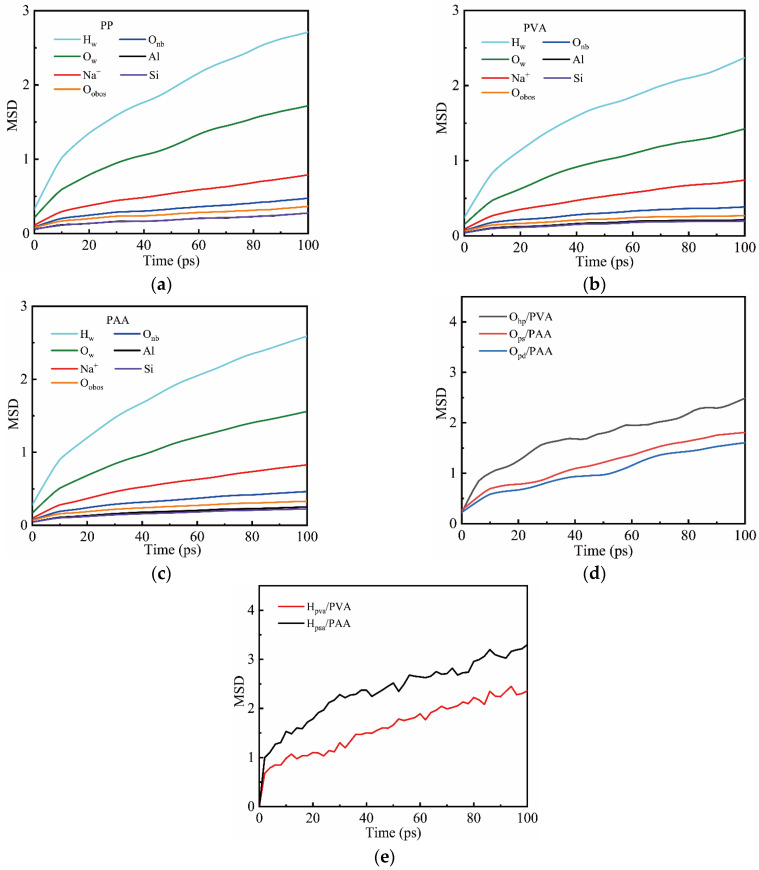
Mean squared displacement of (**a**) PP-NASH; (**b**) PVA-NASH; (**c**) PAA-NASH; (**d**) the polar atoms in fibers; (**e**) H atoms connected to polar atoms (O_obos_ represents the bridging oxygen atoms in NASH).

Figure 6a shows the MSD curves of the NASH matrix atoms in PP-NASH. As the relaxation time increases, compared with other atoms, the MSD curves for both H_w_ and O_w_ atoms show relatively high levels, indicating that water molecules have a relatively strong mobility in the system. In addition, as the charge-compensating cation in the matrix, Na^+^ also exhibits a relatively high mobility in the system. This is because Na^+^ can be distributed in the vacancies of the structure and can interact with the silicon–aluminum tetrahedra through ionic bonds. However, Si, Al, and O_obos_ atoms represent the structural framework, and their MSD curves show extremely low values, revealing the framework stability of NASH. Compared with PP-NASH, the MSD curves for Si, Al, and O_obos_ in PVA-NASH and PAA-NASH also follow a similar pattern (as shown in Figure 6b,c).

Figure 6d,e show the MSD curves of the polar oxygen atoms in fibers and the hydrogen atoms bonded to these polar oxygen atoms. It can be seen that the MSD values of the polar oxygen atoms are lower than those of the hydrogen atoms, and the curves are smoother, indicating that ionic bonds are more stable than hydrogen bonds. The migration rates of O_hp_ and H_pva_ in PVA fibers are higher than those in PAA fibers, indicating that PVA fibers are not fully bonded to the matrix and the stability of the interfacial bonds in PVA fibers is lower than that in PAA fibers.

#### 3.1.4. Interaction Energy

**Figure 7:**
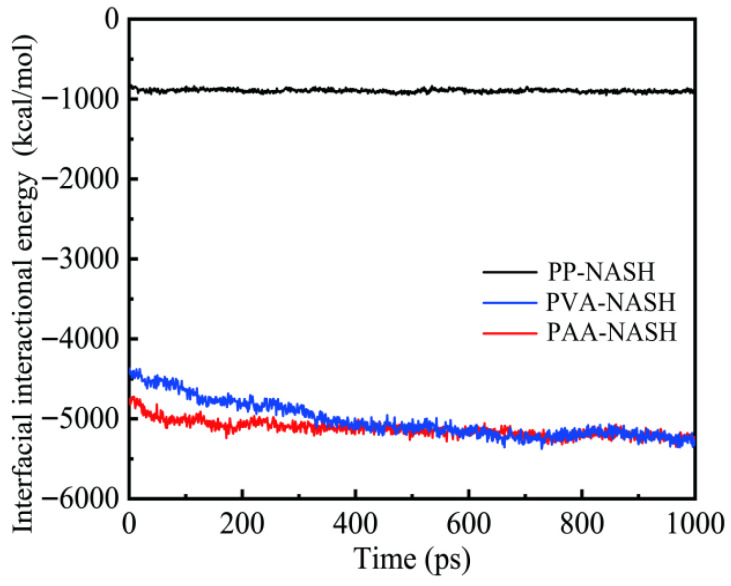
Interfacial interaction energies of PP-NASH, PVA-NASH, and PAA-NASH during the equilibrium process.

The pull-out performance of fibers depends largely on the occurrence of adhesion at the interface. Meanwhile, the interaction energy between interfaces directly reflects the adhesion strength [36]. Therefore, the interaction energy can be a key indicator in evaluating the interfacial adhesion performance [37]. Figure 7 shows the interfacial interaction energies of PP-NASH, PVA-NASH, and PAA-NASH during the equilibrium process. As the relaxation time increases, the interfacial interaction energy tends to be balanced in all the models. However, during the relaxation process, there are obvious differences in the interfacial interaction energy. The interfacial interaction energy in the PP-NASH model reaches equilibrium first. The interfacial interaction energy in the PAA-NASH model tends to reach equilibrium when the relaxation time reaches 200 ps, while that in the PVA-NASH model approaches equilibrium only when the relaxation time reaches 500 ps. After PVA-NASH and PAA-NASH reach equilibrium, the values of their interfacial interaction energies are very close, and both are greater than that of PP-NASH.

### 3.2. Pull-Out Process

#### 3.2.1. Pulling Force and Displacement Curve

**Figure 8:**
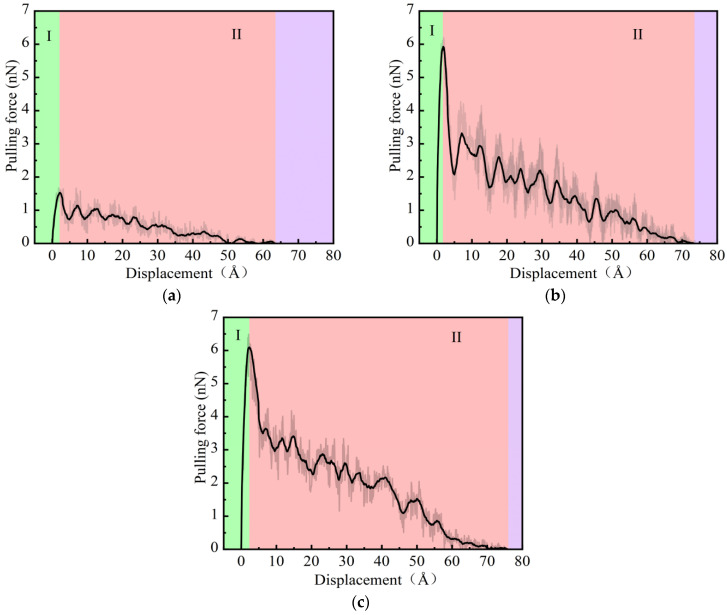
Pulling force and displacement curve under a loading rate of 0.001 Å/fs: (**a**) PP-NASH; (**b**) PVA-NASH; (**c**) PAA-NASH. (Region I represents the debonding stage, and region II represents the pull-out stage).

During the pull-out process, the pulling force reflects the interaction at the interface and the transfer of interface stress. The complete pull-out distance is the distance moved by the fiber end when the fiber is completely separated from the matrix, which can reflect the adhesion performance to a certain extent [38]. Figure 8a shows the pulling force and displacement curve under a loading rate of 0.001 Å/fs. It can be seen that the pull-out process of the PP fiber from the NASH matrix can be divided into two stages: debonding (Region I) and pull-out (Region II). In the debonding stage (Region I), the pulling force of the PP fiber rapidly increases to 1.53 nN and is in an equilibrium state with the interfacial bonding force. In the pull-out stage (Region II), as the displacement increases, the pulling force curve of the PP fiber shows significant fluctuations. This is because during the pull-out process, chemical bonds at the interface are continuously breaking and new chemical bonds are being formed [39]. As shown in Figure 8a–c, comparing the pulling force–displacement curves of three fibers, it can be observed that the pull-out stage (Region II) of PAA-NASH is significantly longer than those of PP-NASH and PVA-NASH. Furthermore, stronger interfacial interactions allow for greater complete pull-out distances (Region I + II). The above discussion indicates an excellent interfacial bonding performance between the PAA fiber and NASH.

**Figure 9:**
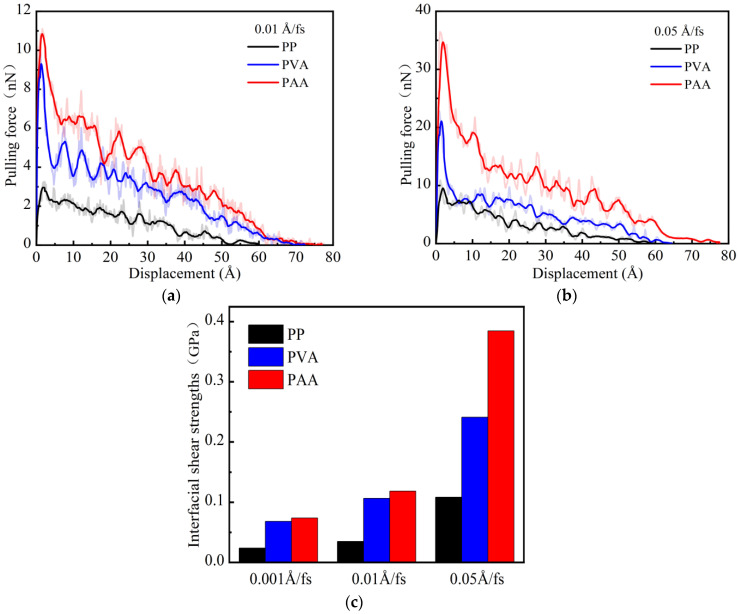
Pulling force and displacement curves under loading rates of (**a**) 0.01 Å/fs and (**b**) 0.05 Å/fs; (**c**) interfacial shear strengths of fibers under different loading rates.

Figure 9a,b show the pulling force and displacement curves under different loading rates. The development trends of the pulling force–displacement curves for different fibers are generally the same, and the ranking relationship of the maximum pulling force values is not affected by the loading rate. In addition, as the loading rate increases, the complete pull-out distance of PVA-NASH decreases from 73.5 Å (0.001 Å/fs) to 64.5 Å (0.05 Å/fs), showing a significant reduction. However, the complete pull-out distances for PAA-NASH and PP-NASH are almost unchanged with the increase in loading rate. This result shows that the influence of the loading rate is greater in PVA-NASH than in the other two models.

Figure 9c shows the interfacial shear strengths of fibers under different loading rates. As the loading rate increases, the interfacial shear strengths of all fibers show an upward trend. The interfacial shear strength increases the most when the loading rate is 0.05 Å/fs. At the low loading rates (0.001 Å/fs and 0.01 Å/fs), the interfacial shear strengths of PVA-NASH and PAA-NASH are similar, and both are higher than that of PP-NASH. At the high loading rate (0.05 Å/fs), the interfacial shear strengths of different fibers with NASH are quite different, and the order is PAA-NASH > PVA-NASH > PP-NASH.

#### 3.2.2. Atomic Displacement in the Matrix

**Figure 10:**
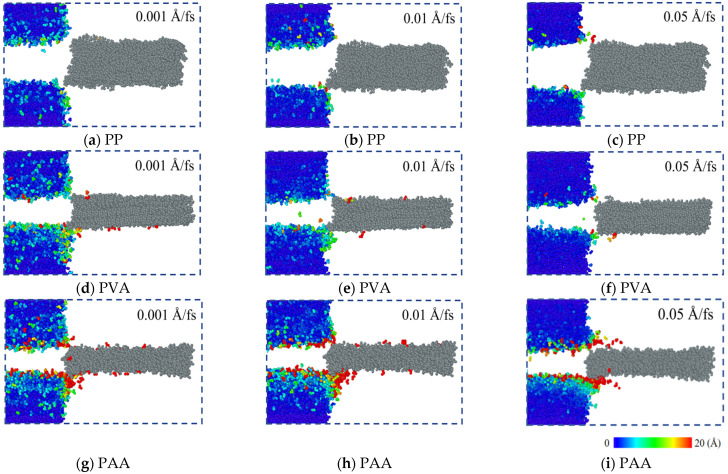
Atomic displacement of (**a**–**c**) PP-NASH, (**d**–**f**) PVA-NASH, and (**g**–**i**) PAA-NASH under different loading rates.

The pulling forces of all fibers are close to zero when the pull-out distance is 70 Å, indicating that all fibers have been basically pulled out. Therefore, the atomic displacement analysis is carried out by taking the displacement nephogram at a pull-out distance of 70 Å as an example. Figure 10a–c show the atomic displacement of PP-NASH under different loading rates. Since there are no polar oxygen-containing functional groups in the PP fiber, the deformation of NASH matrix ends is small. With the loading rate increasing, due to the kinetic energy increase in PP fibers, the end deformation of the matrix shows a slight decreasing trend. Figure 10d–f show the atomic displacement of PVA-NASH under different loading rates. Compared with PP fibers, since PVA fibers have polar oxygen-containing functional groups (hydroxyl groups), the end deformation of the NASH matrix is relatively large. However, as the loading rate increases, since the influence of the loading rate is greater than the anchoring effect of hydroxyl groups, the deformation change at the end of the matrix is significantly reduced. When PVA fibers are pulled out of the matrix, some water molecules are also carried out of the matrix. Figure 10g–i show the atomic displacement of PAA-NASH under different loading rates. The carboxyl functional groups of PAA fibers have stronger polarity than the hydroxyl functional groups of the PVA fiber, and it is easier to form bonds, resulting in greater interfacial interaction energy in PAA-NASH. Therefore, the deformation of the NASH matrix end is greater under loading. As the loading rate increases, the influence of the loading rate is less than the anchoring effect of the carboxyl groups, resulting in there being insignificant changes for the end deformation of the NASH matrix. Meanwhile, when PAA fibers are pulled out of the NASH matrix, more water molecules are carried out.

It is clear that the polar functional groups in fibers can act as anchors embedded in the matrix, resulting in the enhancement of the interfacial bonding. This effect is also often used for the interfacial modification of macroscopic fibers [38]. In addition, fibers with polar functional groups adsorb water molecules in the NASH matrix during the pull-out process, and they will be carried away from the matrix as the displacement increases. The order of the models with regard to the number of water molecules carried away is PAA > PVA > PP. The effectiveness of the anchoring behavior of polar functional groups is also proven by this phenomenon [40].

#### 3.2.3. Chemical Bond Coordination Number

**Figure 11:**
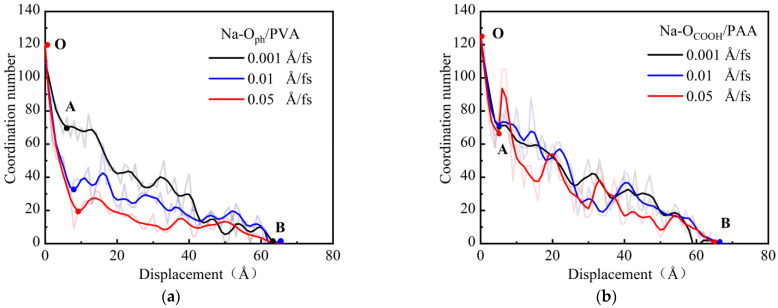
Evolution of the coordination number of Na-O bonds at the interface under different loading rates: (**a**) PVA-NASH; (**b**) PAA-NASH.

To reveal the effect of the influencing mechanism of the loading rate on the anchoring effect of the polar functional groups, we further analyzed the evolution of the Na-O ion bond coordination number during the pull-out process of PVA and PAA fibers at different loading rates [39]. Figure 11 shows the evolution of the coordination number of Na-O bonds at the interface under different loading rates. With the increase in displacement, the change curve for the ionic bond coordination number can be roughly divided into two stages: a steep drop and a slow drop. At the steep drop stage, the continuous breaking of the interfacial chemical bonds leads to a significant decrease in the coordination number. At the slow drop stage, because of the breaking of chemical bonds and the formation of new chemical bonds simultaneously at the interface, the coordination number decreases slowly.

As illustrated in Figure 11a, at the slow drop stage, as the loading rate of PVA fibers increases, since the formation rate of new chemical bonds is lower than the breaking rate of chemical bonds, the coordination number gradually decreases. Furthermore, it can be seen from Figure 11b that, compared with PVA fibers, the carboxyl functional groups of PAA fibers contain more oxygen atoms, and they are beneficial in the formation of new bonds. As the loading rate of PAA fibers increases, the change in the ionic bond coordination number is not obvious at the slow drop stage. The above analysis shows that PVA fibers are more sensitive to the loading rate. The greater the loading rate, the more unstable the hydroxyl group in the structure and the weaker the bonding ability with the matrix, leading to a reduction in the anchoring effect of the PVA fiber on the matrix. This result is consistent with the atomic displacement analysis (Figure 10).

## 4. Conclusions

1. PP fibers become entangled both within and between layers, causing the layered structure to become distorted. Compared with PP fibers, due to PVA and PAA fibers both containing polar oxygen-containing functional groups, there are strong hydrogen-bonding interactions between fibers and the smaller interlayer spacing of fibers after equilibration. However, since the polarity of the hydroxyl group (-OH) in PVA fibers is weaker than that of the carboxyl group (-COOH) in PAA fibers, there is smaller interlayer spacing in PAA fibers than in PVA fibers. These results were further confirmed through the analysis of atomic density distribution.
2. There are both ionic and hydrogen bonds between the fibers with polar functional groups and NASH. Na^+^ ions significantly contribute to the interfacial bonding by forming O_NASH_-Na-O_polymer_ linkages. Hydrogen bonds can be formed between the fibers with polar functional groups and NASH, and these also contribute to improving the interfacial strength to a certain extent. However, ionic bonds are more stable than hydrogen bonds.
3. The pull-out process of fibers from NASH can be divided into a debonding and a pull-out stage. As the loading rate increases, the interfacial shear strengths of all fibers show an upward trend, and the strength increases the most when the loading rate is 0.05 Å/fs. At the low loading rates, the interfacial shear strengths of PVA-NASH and PAA-NASH are similar, and both are higher than that of PP-NASH. At the high loading rates, the interfacial shear strengths of different fibers vary greatly.
4. Compared with PP fibers, there are polar oxygen-containing functional groups (hydroxyl groups) in PVA fibers, and the end deformation of NASH is relatively large. Since the carboxyl functional groups in PAA fibers have stronger polarity than the hydroxyl functional groups in PVA fibers, the end deformation on NASH is greater. In addition, as the loading rate increases, since the influence of the loading rate is greater than the anchoring effect of the hydroxyl groups, the end deformation change in NASH is significantly reduced. With the loading rate increasing, the influence of the loading rate is less than the anchoring effect of the carboxyl groups, resulting in there being insignificant changes in the end deformation of NASH.

## Data Availability

The original contributions presented in the study are included in the article, further inquiries can be directed to the corresponding author.
